# Microbiome-Derived Short-Chain Fatty Acids and Tryptophan Metabolites in Children with Autism Spectrum Disorder: A Stool–Urine Multi-Omics Analysis

**DOI:** 10.3390/ijms27093988

**Published:** 2026-04-29

**Authors:** Joško Osredkar, Teja Fabjan, Uroš Godnov, Maja Jekovec-Vrhovšek, Damjan Osredkar, Petra Finderle, Kristina Kumer, Maša Zorec, Lijana Fanedl, Gorazd Avguštin

**Affiliations:** 1 Institute of Clinical Chemistry and Biochemistry, University Medical Centre Ljubljana, Zaloška Cesta 2, 1000 Ljubljana, Slovenia; josko.osredkar@kclj.si (J.O.); petra.finderle@kclj.si (P.F.); kristina.kumer@kclj.si (K.K.); 2 Faculty of Pharmacy, University of Ljubljana, Aškerčeva 7, 1000 Ljubljana, Slovenia; 3 The Faculty of Mathematics, Natural Sciences and Information Technologies, University of Ljubljana, Glagoljaška Ulica 8, 6000 Koper, Slovenia; uros.godnov@upr.si; 4 Center for Autism, Unit of Child Psychiatry, University Medical Centre Ljubljana, Zaloška Cesta 2, 1000 Ljubljana, Slovenia; maja.jekovec@kclj.si; 5 Department of Child, Adolescent and Developmental Neurology, Division of Pediatrics, University Medical Centre Ljubljana, Zaloška Cesta 2, 1000 Ljubljana, Slovenia; damjan.osredkar@kclj.si; 6 Faculty of Medicine, University of Ljubljana, Vrazov Trg 2, 1000 Ljubljana, Slovenia; 7 Department of Microbiology, Biotechnical Faculty, University of Ljubljana, Groblje 3, 1230 Domžale, Slovenia; masa.zorec@bf.uni-lj.si (M.Z.); lijana.fanedl@bf.uni-lj.si (L.F.)

**Keywords:** autism spectrum disorder, gut microbiota, short-chain fatty acids, tryptophan metabolism, indole metabolites, kynurenine pathway, epigenetics, microbiome–epigenome interaction, metabolomics, pediatrics

## Abstract

Autism spectrum disorder (ASD) has been associated with alterations in the gut microbiota and its metabolites, particularly short-chain fatty acids (SCFAs) and microbiota-derived tryptophan catabolites, which may influence neurodevelopment through immune and epigenetic mechanisms. We investigated whether stool SCFAs and tryptophan-pathway metabolites differ between children with ASD and typically developing controls, and whether these metabolites associate with ASD severity and systemic biochemical signatures. In this cross-sectional study, we analyzed stool samples from 229 children (160 with ASD, 69 controls) with complete SCFA and tryptophan-metabolite data, while urine metabolomics data were available for a subset and were used for exploratory stool–urine integration analyses. Children with ASD and controls were similar in age, but the ASD group had a higher proportion of males. Absolute concentrations of individual SCFAs, total SCFAs, and derived indices were broadly comparable between groups; nominal differences in propionate/acetate ratio and caproate did not remain significant after false discovery rate correction. Similarly, stool tryptophan-pathway metabolites reported as ng/a.u. based on the NanoDrop-derived proxy (tryptophan, kynurenine, indole-3-acetic, indole-3-lactic, indole-3-propionic, indole-3-aldehyde, N-acetyl-tryptophan, serotonin, melatonin, tryptamine) and functional ratios (kynurenine/tryptophan, indole-derived/tryptophan, serotonin/tryptophan) showed no robust ASD–control differences; N-acetyl-tryptophan was nominally higher in ASD but did not survive multiple-testing correction. In the ASD subgroup with available Childhood Autism Rating Scale (CARS) data (n = 34), SCFA and tryptophan indices showed only weak, non-significant correlations with global ASD severity. In contrast, correlation analyses revealed two coherent metabolic modules, i.e., an SCFA block with very strong internal correlations among individual SCFAs and total SCFAs and a tryptophan block with strong correlations between metabolites and their normalized ratios, while cross-module correlations were modest. These results indicate that stool SCFA and microbiota-derived tryptophan profiles do not robustly distinguish ASD from controls in this cohort, but they form stable metabolic modules compatible with microbiome–epigenome frameworks.

## 1. Introduction

Autism spectrum disorder (ASD) is a heterogeneous neurodevelopmental condition characterized by persistent deficits in social communication and interaction, alongside restricted, repetitive patterns of behavior and interests, frequently accompanied by cognitive, behavioral, and medical comorbidities [1]. Increased awareness, broader diagnostic criteria, and advancements in case detection have all contributed to its rising reported prevalence over the past few decades. However, there has also been an expansion of research in possibly modifiable biological and environmental risk factors [2,3,4,5,6]. Many individuals with ASD exhibit gastrointestinal (GI) symptoms, dietary selectivity, sleep problems, and immune dysregulation, which has focused attention on the bidirectional communication within the gut–brain axis and the potential contribution of the intestinal microbiota and its metabolites to ASD pathophysiology [7,8,9,10,11].

Some studies indicate that children with ASD show differences in gut microbiota composition compared with typically developing peers, although findings are not entirely consistent across cohorts, methods, and geographic regions [12,13,14,15,16,17]. The idea that the gut microbiome causally contributes to autism has gained currency in the scientific literature and popular press. Support for this hypothesis comes from three lines of evidence: human observational studies, preclinical experiments in mice, and human clinical trials. Kevin J. Mitchell has argued that claims about a causal role of the gut microbiome in autism remain limited by conceptual and methodological flaws [18]. Systematic reviews and meta-analyses suggest altered alpha and beta diversity and shifts in the relative abundance of specific taxa, with repeated reports of changes in *Bacteroidetes*, *Firmicutes*, *Proteobacteria*, and *Actinobacteria*, as well as genera such as *Clostridium*, *Bacteroides*, and *Sutterella* in ASD [13,15,19,20,21,22]. More recent work has extended these observations to the oral microbiome and to integrative multi-omics datasets, in which ASD-associated microbial changes co-occur with alterations in metagenomic functional capacity, metaproteomes, and metabolite profiles, implicating pathways related to neurodevelopment, immune regulation, and energy metabolism [23,24,25]. Despite this progress, there is substantial heterogeneity between studies, and it remains unclear which microbiome features, if any, are robust and causally relevant across the diverse ASD spectrum.

Short-chain fatty acids (SCFAs) are among the most intensively studied microbiota-derived metabolites in ASD [21,26,27,28,29]. Produced by bacterial fermentation of dietary fibers and resistant starches, SCFAs such as acetate, propionate, and butyrate regulate gut barrier integrity, mucus production, and motility, modulate innate and adaptive immune responses, and influence host energy balance [30,31,32,33,34]. SCFAs can reach the systemic circulation and the central nervous system, where they signal through G-protein-coupled receptors and also exert epigenetic effects by modulating histone acetylation: butyrate and, to a lesser extent, propionate can inhibit histone deacetylases (HDACs) and/or activate histone acetyltransferases such as p300, leading to changes in chromatin accessibility and gene expression in epithelial, immune, and neural cells [30,35,36]. Epigenetic dysregulation, including altered histone modifications and HDAC activity, is increasingly recognized as a key mechanism by which environmental and metabolic factors may converge on ASD-related gene networks [37,38].

Preclinical models support a link between SCFAs and ASD-like phenotypes. In rodent paradigms, intragastric or intracerebroventricular administration of propionic acid at supraphysiological doses induces behavioral changes reminiscent of ASD, including social impairments, repetitive behaviors, and anxiety-like phenotypes, together with neuroinflammatory and mitochondrial alterations [39]. These data have given rise to the hypothesis that increased luminal or systemic propionate or altered SCFA profiles could contribute to ASD pathogenesis in susceptible individuals [26,28,40]. Human studies of fecal SCFAs in ASD, however, have yielded mixed results: some report elevated concentrations of acetate, propionate, butyrate, or valerate in ASD, particularly in children with GI symptoms or specific dietary patterns, whereas others find only modest or no differences compared with controls [26,41,42,43]. Meta-analysis by Yang et al. [44] provides evidence for altered SCFA profiles in ASD, specifically elevated valeric and hexanoic acids and a consistent signal for isobutyric acid, suggesting gut microbial dysbiosis involving distinct metabolic pathways. The significant heterogeneity and sample-source-dependent effects highlight the complexity of the gut–brain axis in ASD and underscore the need for future research with standardized protocols and longitudinal designs to clarify the role of SCFAs [44]. Differences in cohort selection, age, diet, GI comorbidities, sample processing, and analytical techniques likely contribute to these discrepancies, and there is a need for larger, methodologically harmonized studies to clarify whether fecal SCFAs represent robust ASD markers or primarily reflect context-specific gut ecology.

A second major axis of microbiota–host interaction involves tryptophan metabolism [45,46,47,48,49]. Tryptophan is a precursor for host serotonin and kynurenine pathways, but is also metabolized by intestinal bacteria into a diverse array of indole derivatives, including indole-3-acetic acid, indole-3-lactic acid, indole-3-propionic acid, indole-3-aldehyde, and tryptamine [50,51]. These microbial tryptophan catabolites, together with kynurenine and downstream metabolites, act as ligands for the aryl hydrocarbon receptor (AhR) and other nuclear receptors, thereby modulating transcriptional programs that regulate intestinal barrier function, mucosal immune tone, oxidative stress responses, and neuroinflammation [52,53,54,55]. Reviews on tryptophan metabolism in neurodevelopment emphasize that both the host kynurenine branch and the microbiota-derived indole branch can influence synaptic plasticity, microglial maturation, and neuronal excitability, and that dysregulation of these pathways may contribute to neurodevelopmental disorders such as ASD [56,57,58].

Clinical studies support alterations in tryptophan metabolism in ASD, particularly at the systemic level. Several reports describe shifts toward kynurenine pathway activation, changes in serotonin availability, and altered levels of neuroactive kynurenine metabolites, which have been linked to immune activation and glutamatergic dysregulation in ASD [58,59,60,61]. More recently, multimodal studies combining functional MRI with fecal metabolomics have shown that abundances of gut-derived tryptophan metabolites, including indoles and serotonin, correlate with task-related brain activity in regions such as the insula and anterior cingulate cortex and with ASD symptom severity, providing direct evidence of gut–brain coupling via tryptophan metabolites in ASD [62]. Nevertheless, human data on stool-level profiles of microbial tryptophan metabolites in ASD remain limited, and it is not yet clear to what extent these stool signatures consistently distinguish ASD from typical development across cohorts and ages.

Both SCFAs and tryptophan-pathway metabolites occupy central positions within broader microbiome–epigenome frameworks. The MIAOME (Microbiome Impact on the Host Epigenome) concept systematically catalogs microbiota-derived metabolites (MDMs), components (MDCs), and secreted proteins (MSPs) according to their effects on host epigenetic mechanisms, including DNA methylation, histone modifications, and non-coding RNAs [63]. Within this framework, SCFAs such as butyrate and propionate are recognized as potent modulators of histone acetylation, while tryptophan-derived indoles and kynurenine-pathway metabolites modulate transcriptional programs via AhR and other receptors, thereby linking microbial composition and metabolic activity to long-term changes in host gene expression and disease susceptibility [64]. In parallel, epigenetic studies in ASD have identified widespread alterations in DNA methylation and histone marks in genes involved in synaptic function, neuronal signaling, and immune pathways, supporting a model in which genetic susceptibility interacts with epigenetically active environmental and metabolic signals during critical developmental windows [38,65,66,67].

Despite strong mechanistic plausibility, relatively few human studies have jointly quantified stool SCFAs and microbiota-derived tryptophan metabolites in well-characterized pediatric ASD cohorts, and even fewer have integrated these stool measures with systemic metabolomics and standardized clinical severity scales. Most available work has examined microbiota composition, SCFAs, or tryptophan metabolites separately, often in small samples and without systematic assessment of the internal correlation structure of these metabolites or their organization into metabolic “modules” [49,68,69,70]. Emerging integrative multi-omics analyses in ASD indicate that microbial compositional changes are accompanied by shifts in microbial pathways and metabolite profiles, and that certain metabolites may mediate associations between microbial genomic variants and ASD diagnosis or traits, but these studies rarely provide detailed SCFA and indole panels in stool in parallel with clinical measures [71,72,73,74]. As a result, it remains unclear whether stool SCFA and tryptophan profiles in school-age children with ASD are meaningfully different from those of typically developing peers, whether they correlate with global ASD severity, and how they cluster into correlated metabolic axes that may reflect underlying microbiome–host programming.

Therefore, the specific aim of this study was to characterize stool short-chain fatty acid and microbiota-derived tryptophan metabolite profiles, and their internal correlation structure, in a large cohort of school-age children with autism spectrum disorder and typically developing controls.

We further aimed to evaluate whether these metabolites and their key ratios differ between groups or associate with ASD severity and systemic urine metabolomic markers, thereby testing the hypothesis that microbiome-derived metabolic modules relevant to epigenetic programming are altered in pediatric ASD.

## 2. Results

### 2.1. Study Population

In total, 229 children (160 with ASD and 69 typically developing controls) had complete stool SCFA and tryptophan-metabolite data and were included in the primary analysis cohort (Table 1). Age distributions were similar between groups (median 8.5 years in ASD vs. 9.6 years in controls, *p* = 0.354), whereas the ASD group showed a higher proportion of boys (77.5% vs. 56.5%, *p* = 0.002). CARS scores were available for 34 ASD participants (median 38 [30,31,32,33,34,35,36,37,38,39,40,41,42,43,44]). Urine metabolomics data were available in 134 ASD (83.8%) and 60 controls (87.0%), enabling exploratory analyses of stool–urine associations. Because the main focus of this manuscript was stool-derived microbiome-related metabolites, urine metabolomics were not analyzed here as a separate primary outcome but were retained to document multi-omics coverage and support exploratory cross-matrix analyses.

### 2.2. Stool Short-Chain Fatty Acids

SCFA concentrations and derived indices were broadly similar between groups (Table 2). Acetate was 49.84 mM [36.57–62.82] in ASD and 48.56 mM [37.08–56.03] in controls (*p* = 0.285). Propionate was 12.68 mM [8.98–17.96] vs. 14.06 mM [10.74–17.23] (*p* = 0.194), and butyrate was 13.98 mM [9.97–24.59] vs. 13.95 mM [9.72–18.60] in ASD and controls, respectively (*p* = 0.269). Total SCFAs did not differ significantly (87.73 mM [63.72–114.07] vs. 85.30 mM [65.77–101.62]; *p* = 0.360).

Among derived indices, the propionate/acetate ratio was modestly lower in ASD (0.27 [0.22–0.33]) than in controls (0.30 [0.25–0.36]; *p* = 0.028), but this difference did not remain significant after false discovery rate correction (q = 0.235). Caproate concentrations showed a similar nominal trend (*p* = 0.043, q = 0.235). No SCFA or ratio showed a robust ASD–control difference when accounting for multiple comparisons.

### 2.3. Stool Tryptophan-Pathway Metabolites

Stool tryptophan metabolites, expressed as ng/a.u. based on the NanoDrop-derived proxy, were also largely comparable between ASD and controls (Table 3). Tryptophan levels were 2.64 ng/a.u. [1.57–3.71] in ASD and 2.09 [1.68–3.16] in controls (*p* = 0.221). Kynurenine concentrations were nearly identical between groups (2.17 [1.46–3.84] vs. 2.06 [1.35–4.34]; *p* = 0.994). Indole-3-acetic, -lactic, -propionic, and -aldehyde levels, as well as serotonin, melatonin, tryptamine, and N-acetyl-tryptophan levels, showed overlapping distributions between ASD and controls.

N-acetyl-tryptophan showed a nominally higher median in ASD than in controls (5.16 [2.71–8.52] vs. 3.83 [1.62–6.46]; *p* = 0.022), but this difference did not remain significant after FDR correction (q = 0.314). Ratios capturing pathway balance—KYN/TRP, IPA/TRP, IAA/TRP, SER/TRP, and an aggregate Indole/TRP ratio—were also similar between groups, with all q values ≥ 0.735. Thus, microbiota-derived tryptophan metabolites and their key ratios did not show statistically robust ASD–control differences in this cohort.

### 2.4. Associations with Clinical Severity

Within the ASD group, we examined correlations between selected stool metabolite indices and CARS scores in the subset of children with available severity data (n = 34). None of the SCFA or tryptophan variables showed a meaningful correlation with CARS, and all *p* values were non-significant (Table 4). Specifically, correlations between CARS and selected SCFA indices (butyrate, propionate, butyrate/acetate, propionate/acetate) were weak (all |ρ| ≤ 0.08, all *p* > 0.63), and correlations between CARS and tryptophan-pathway ratios (KYN/TRP, IPA/TRP, Indole/TRP, SER/TRP) were similarly small (all |ρ| ≤ 0.16, all *p* > 0.37). No meaningful linear trend between stool metabolite patterns and global ASD severity could be detected, although these analyses were limited by the modest number of ASD children with CARS scores.

### 2.5. Correlation Structure of SCFAs and Tryptophan Metabolites

We next explored the correlation structure across all SCFA- and tryptophan-related variables in the full cohort of 229 children. The Spearman correlation matrix revealed two coherent metabolic modules: an SCFA block with strong internal correlations among acetate, propionate, butyrate, branched-chain SCFAs, and total SCFAs (e.g., acetate vs. total SCFAs ρ = 0.94, *p* ≪ 10^−20^; butyrate vs. total SCFAs ρ = 0.88, *p* ≪ 10^−20^) and a tryptophan block with strong correlations between metabolites and their normalized ratios (e.g., IAA vs. IAA/TRP ρ = 0.81, IPA vs. IPA/TRP ρ = 0.79, SER vs. SER/TRP ρ = 0.79; all *p* ≪ 10^−49^).

**Figure 1:**
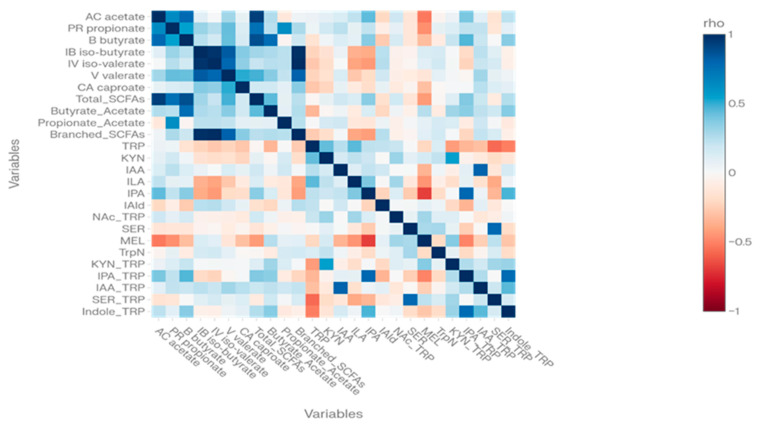
Heatmap of Spearman correlations between stool SCFA indices and tryptophan-pathway metabolites (n = 229). Positive correlations are shown in red and negative correlations in blue, with color intensity proportional to |rho|. Two main correlation modules corresponding to SCFAs and tryptophan metabolites are evident. SCFAs, short-chain fatty acids; TRP, tryptophan; KYN, kynurenine; IAA, indole-3-acetic acid; ILA, indole-3-lactic acid; IPA, indole-3-propionic acid; IAld, indole-3-aldehyde; NAc_TRP, N-acetyl-tryptophan; SER, serotonin; MEL, melatonin; TrpN, tryptamine.

Cross-module correlations between SCFA indices and tryptophan variables were generally modest, with no pair exceeding |ρ| = 0.5, indicating that SCFA and tryptophan pathways form partially independent metabolic modules in this dataset. This apparent partial separation should be interpreted cautiously, because the SCFA and tryptophan panels were generated using different analytical workflows and normalization strategies, which may reduce the magnitude of observed cross-module correlations. A heatmap of the full Spearman correlation matrix (11 SCFA-related variables and 15 tryptophan-related variables) visually highlights these two blocks and their internal structure (Figure 1).

Together, these findings show that the cohort exhibits coherent SCFA and tryptophan metabolic modules compatible with known microbiome-derived programming pathways, even though their absolute levels and ratios are not substantially shifted in ASD compared with controls.

## 3. Discussion

This study used an integrated stool–urine multi-omics approach in a relatively large, well-characterized pediatric cohort to examine whether microbiome-derived short-chain fatty acids (SCFAs) and tryptophan-pathway metabolites differ between children with autism spectrum disorder (ASD) and typically developing controls, and whether these metabolites are associated with clinical severity. Our primary findings are that (i) absolute concentrations and key ratios of stool SCFAs and microbiota-derived tryptophan metabolites do not show robust cross-sectional differences between ASD and controls at school age after correction for multiple testing, (ii) these metabolites do not correlate meaningfully with global ASD severity (CARS) in the ASD subgroup with available data, and (iii) SCFAs and tryptophan metabolites form distinct, internally coherent metabolic modules, suggesting stable gut metabolic axes that are compatible with microbiome–epigenome interaction frameworks but not substantially shifted in this cohort.

### 3.1. SCFAs in ASD: Reconciling Inconsistent Human Data

SCFAs are among the most intensively discussed microbiota-derived metabolites in ASD because of their ability to influence gut barrier function, immune responses, and brain physiology. Acetate, propionate, and butyrate are produced by bacterial fermentation of dietary fibers and resistant starches, and they signal through G-protein-coupled receptors and epigenetic mechanisms, including modulation of histone acetylation [32,35,75,76]. In rodent models, intracerebroventricular or intragastric administration of propionic acid at supraphysiological doses induces ASD-like behaviors, neuroinflammation, and alterations in glial and neuronal markers, fueling the hypothesis that increased luminal or systemic propionate might contribute to ASD pathophysiology [77,78,79].

Human studies, however, have yielded heterogeneous results. Several small case–control cohorts reported higher fecal levels of specific SCFAs (e.g., acetate, propionate, butyrate, valerate) in ASD compared with controls, often accompanied by gut dysbiosis and gastrointestinal (GI) symptoms [21]. For instance, a Chinese study found altered gut microbiota composition and associations between SCFA production and constipation in children with ASD, although not all individual SCFAs differed significantly between groups [43]. A more recent study integrating microbiota and SCFA measures in ASD also suggested dysbiosis with predicted SCFA imbalances, but direct SCFA quantification was limited and conclusions about metabolite levels remained tentative [26]. Conversely, other cohorts did not observe consistent SCFA differences, or reported changes confined to subgroups defined by GI symptoms or diet stool consistency, and technical factors on SCFA measurements [80,81].

Within this context, our finding of broadly similar acetate, propionate, butyrate, and total SCFA levels between ASD and controls aligns with studies that report either no major shifts in SCFAs or only modest differences when controlling for confounders. We observed nominally lower propionate/acetate ratios and higher caproate in ASD, but these did not survive FDR correction, indicating that any group differences are small relative to inter-individual variability. Importantly, our cohort was not selected for severe GI pathology; thus, our data suggest that in relatively unselected school-age ASD populations, gross fecal SCFA abnormalities are not a consistent feature and are unlikely to serve as robust standalone biomarkers. This does not negate the possibility that specific ASD subtypes, younger developmental windows, or individuals with pronounced GI symptoms could exhibit more marked SCFA alterations, as some prior work indicates [26,41,82,83].

### 3.2. Tryptophan and Indole Metabolites: Complex Signaling with Modest Group Differences

Tryptophan metabolism represents another key microbiota–host interface. Beyond host serotonin and kynurenine pathways, gut bacteria convert tryptophan into diverse indole derivatives (e.g., indole-3-acetic, -lactic, -propionic, -aldehyde) and tryptamine, many of which modulate the AhR and other transcriptional regulators, influencing barrier integrity, immune tone, and neuroinflammation. The MIAOME framework systematically catalogs microbiota-derived metabolites—including SCFAs and tryptophan catabolites—as upstream effectors of DNA methylation, histone modifications, and non-coding RNA expression in host tissues, emphasizing their potential role in long-term epigenetic programming [63].

Clinically, altered systemic tryptophan metabolism has been reported in ASD. Serum or plasma studies have described reduced tryptophan and serotonin availability, shifts toward kynurenine pathway activation, and increased downstream metabolites such as quinolinic acid, linking immune activation to glutamatergic neurotransmission [61,84,85,86]. More recently, an imaging–metabolomics study showed that fecal tryptophan metabolites, particularly indoles, were associated with altered brain activity in insular and cingulate regions and with ASD symptom severity, strongly implicating gut-derived indoles in ASD-related brain–behavior changes [62].

Our stool data complement and partially contrast these findings. When normalized to nucleic acid, absolute levels of tryptophan, kynurenine, multiple indoles (IAA, ILA, IPA, IAld), serotonin, melatonin, tryptamine, and the derived ratios (KYN/TRP, IPA/TRP, IAA/TRP, SER/TRP, Indole/TRP) did not differ significantly between ASD and controls after FDR correction, although N-acetyl-tryptophan showed a nominally higher median in ASD. This suggests that, in this cohort, gross alterations in the stool indole signature are not a universal hallmark of ASD, in contrast to some reports where indole metabolites showed stronger group separation or links to brain activity and symptom scores [62,87].

Several explanations are plausible. First, our cohort is clinically heterogeneous and school-aged, whereas some studies focus on narrower phenotypic or developmental windows, and there is increasing evidence that microbiome-related metabolic effects on neurodevelopment may be strongest in early life. Accordingly, the relative metabolic stability observed here may represent a post-critical-window state rather than evidence against earlier developmental dysbiosis. Longitudinal sampling beginning in infancy or early childhood will be needed to determine whether microbiome-derived metabolic disturbances are more prominent before the school-age period examined in the present study. Second, the use of nucleic acid normalization in our study emphasizes metabolite production per microbial biomass, which may capture different aspects of microbial metabolism than concentration per stool weight; some prior studies use other normalizations or semi-quantitative methods, complicating direct comparison [88,89]. This distinction is important because the partial separation of stool metabolite patterns across studies may reflect not only biological heterogeneity, but also differences in sample normalization, matrix characteristics, and analytical workflows. Future studies should directly compare NanoDrop-based normalization with dry-weight and other compositional approaches in the same cohort. Third, differences in diet, geography, and analytical platforms (including panel composition and limits of detection) can influence measured metabolite profiles and may partly account for discrepancies across cohorts.

Taken together, our findings are consistent with the idea that microbiota-derived tryptophan metabolism is mechanistically relevant to ASD, as supported by systemic and brain-correlational studies, but indicate that cross-sectional stool concentrations and simple ratios alone may not robustly distinguish ASD from controls in all settings [56,62]. Rather, these metabolites may exert their effects via context-dependent, circuit-specific interactions (e.g., on AhR in barrier and immune cells, or through downstream kynurenine metabolites in the brain) that are not fully captured by bulk stool measurements.

### 3.3. CARS Severity and the Limits of Cross-Sectional Metabolite–Phenotype Correlations

We did not observe meaningful correlations between stool SCFA or tryptophan indices and CARS scores within the ASD group, with all Spearman coefficients small (|ρ| ≤ 0.16) and non-significant. This stands in contrast to some reports where fecal indoles or SCFAs correlated with symptom severity or behavioral scales, including studies showing associations between indole derivatives and ASD severity or alexithymia, and between butyrate and social responsiveness or sleep measures [21,41,62]. The discrepancy likely reflects several factors: our modest sample size for severity analyses (n = 34 with CARS), differences in the specific behavioral instruments used, and potential non-linear or time-lagged relationships between gut metabolites and clinical phenotypes.

Moreover, global severity scores such as CARS may be less sensitive to metabolite-linked dimensions (e.g., sensory sensitivity, anxiety, GI symptom burden) than more targeted scales, and our study did not stratify ASD children by GI symptoms or dietary patterns, which have been shown to modulate SCFA and metabolite associations with behavior. Thus, while our negative correlation findings argue against simple monotonic relationships between stool metabolites and overall ASD severity in this cohort, they do not exclude more nuanced or domain-specific links that would require larger, deeply phenotyped samples for detection.

### 3.4. Metabolic Modules and Microbiome–Epigenome Frameworks

A key contribution of our study is the characterization of the internal correlation structure of stool SCFAs and tryptophan metabolites. We observed two distinct, coherent modules: an SCFA block with very strong correlations among individual SCFAs, branched-chain SCFAs, and total SCFAs and a tryptophan block with strong correlations between parent metabolites and their nucleic acid-normalized ratios (e.g., IAA vs. IAA/TRP, IPA vs. IPA/TRP, SER vs. SER/TRP, and IPA/TRP vs. the aggregate Indole/TRP ratio).

This modular structure is in line with the broader concept that microbiota-derived metabolites act in coordinated “axes” or “programmes” that influence host physiology and epigenetics. The MIAOME framework explicitly groups microbiota-derived metabolites (MDMs), components (MDCs), and secreted proteins (MSPs) according to their epigenetic effects, including modulation of histone acetylation, DNA methylation, and non-coding RNA expression [63,90]. SCFAs such as butyrate and propionate can increase histone acetylation through effects on p300/CBP acetyltransferase activity and HDAC inhibition, thereby altering gene expression patterns in epithelial, immune, and neural cells [91,92]. Indole derivatives and kynurenine-pathway metabolites, in turn, act through AhR and other receptors to regulate transcriptional programs linked to barrier function, immune tolerance, and inflammatory responses [91,93].

Our data do not provide direct evidence of epigenetic modifications, but the presence of stable SCFA and tryptophan modules in both ASD and controls is compatible with the notion that these metabolic axes constitute a conserved “toolbox” through which the microbiome can influence host epigenome and physiology. The absence of large ASD–control shifts in these modules at school age may indicate that, in many children, the microbiome–metabolite system has converged toward a broadly similar state despite earlier-life perturbations, or that critical epigenetic programming events linked to these metabolites occurred during earlier developmental windows not captured by our cross-sectional sampling. Alternatively, more subtle differences may exist at the level of microbial genomic variants, specific enzyme isoforms, or metabolite fluxes that are not reflected in steady-state stool concentrations; recent large-scale multi-omics studies highlight microbial genomic variation and metabolite mediation as important layers in ASD-associated dysbiosis [23,25,94]. At the same time, this partial modular separation should not be equated with complete biological independence. Differences in assay platform, extraction protocol, signal dynamic range, and normalization strategy may also contribute to attenuated cross-module correlations and should be considered when interpreting these patterns.

### 3.5. Strengths, Limitations, and Implications for Future Research

The strengths of our study include the relatively large sample size for a metabolomics study in ASD (229 children), the strict requirement of complete SCFA and stool tryptophan-panel data, and the integration of these stool measures with urine metabolomics and detailed clinical information. The use of nucleic acid normalization for tryptophan metabolites and derived ratios provides a robust measure of metabolite production relative to microbial biomass, and the comprehensive correlation analyses allowed us to move beyond single metabolite comparisons to a more system-level view of metabolic modules.

However, several limitations warrant consideration. First, our design is cross-sectional; we cannot address temporal dynamics or early-life programming effects, which are central to many microbiome–neurodevelopment hypotheses. Second, although our total cohort is sizeable, the subset with CARS scores is modest (n = 34), limiting power to detect metabolite–severity correlations. Third, we did not incorporate detailed dietary data, GI symptom scores, or antibiotic/probiotic exposures, all of which can significantly influence both microbiota composition and metabolite production. Fourth, while we document availability of 16S/shotgun sequencing data, microbiome compositional and functional profiles were not integrated into the present analyses; thus, we cannot link specific taxa, pathways, or microbial genomic variants to the observed metabolite modules. Another limitation is that 16S amplicon sequencing data were available only in a subset of participants and were markedly imbalanced between groups, which limited the interpretability of any descriptive microbiome-composition analysis within the present dataset. For this reason, microbial taxonomic analyses were not included here and should be addressed in future integrative studies specifically designed for matched microbiome–metabolite comparisons.

An additional limitation is that the control group included both unrelated typically developing children and siblings of children with ASD. Although this approach improved recruitment feasibility and age matching, sibling controls may share environmental, dietary, household, and familial microbiome-related exposures with ASD participants, potentially attenuating between-group differences and limiting external generalizability.

Finally, our negative or modest findings should not be overinterpreted as evidence against microbiome involvement in ASD. Instead, they refine the field by suggesting that, in unselected school-age cohorts, stool SCFA and tryptophan metabolite levels and simple ratios alone may not serve as robust diagnostic markers, and that the impact of these metabolites on ASD may be mediated by developmental timing, specific host–microbe interactions, and molecular mechanisms not captured by bulk stool profiles.

The term “stool–urine multi-omics” in the title reflects the integrated design and availability of both biological matrices in the cohort; however, the present report is centered on stool-derived microbiome-related metabolites, while urine data serve a supporting exploratory role and are discussed only to that extent.

Future research should prioritize longitudinal, multi-omics designs that combine microbiome composition (including strain-level and genomic-variant information), stool and systemic metabolites, host epigenetic marks, and detailed phenotyping from early life onward. Such studies could clarify whether early perturbations in SCFA and tryptophan modules imprint lasting epigenetic and neuroimmune changes, and identify subgroups of ASD in which microbiome-derived metabolic signatures are most pronounced and therapeutically actionable. Interventional trials using dietary fiber modulation, targeted probiotics, or metabolite mimetics, ideally with embedded epigenetic and neuroimaging readouts, will be essential to move from correlation to causation and to test whether manipulating these metabolic axes can improve clinically relevant outcomes.

## 4. Materials and Methods

### 4.1. Study Design and Participants

This was a cross-sectional observational study including children with autism spectrum disorder (ASD) and typically developing controls recruited at a single tertiary center. Children with ASD were diagnosed according to standard clinical criteria by experienced child psychiatrists and psychologists, based on DSM-5-compatible assessments and multidisciplinary evaluation. Additional behavioral ratings were based on a standardized behavior classification for children with ASD, developed by the local educational authority to provide additional school support and [95,96] Childhood Autism Rating Scale (CARS). The CARS questionnaire was filled out by all parents together with a psychologist. Controls were typically developing siblings and unrelated children without a history of ASD or other major neurodevelopmental disorders, recruited from the same catchment area. Exclusion criteria for both groups included known monogenic syndromes, major chromosomal abnormalities, severe chronic somatic disease, and acute gastrointestinal infection at the time of sampling.

In total, 232 stool samples were collected; one adult participant (AS 14) was excluded a priori, leaving 231 pediatric samples. A complete stool short-chain fatty acid (SCFA) profile and stool tryptophan-metabolite panel were available for 229 children, who comprised the primary analysis cohort (160 ASD, 69 controls). Demographic and clinical data (age, sex, clinical classification, diagnosis codes, and Childhood Autism Rating Scale [CARS] scores where available) were extracted from clinical records and linked to laboratory data via unique study identifiers.

### 4.2. Ethical Approval

The study was conducted in accordance with the Declaration of Helsinki and approved by National Medical Ethics Committee (0120-201/2016-2 KME 484 78/03/16 dated 3 February 2021). Written informed consent was obtained from parents or legal guardians of all participating children, and assent was obtained from children when appropriate according to age and cognitive status.

### 4.3. Sample Collection and Processing

Stool samples were collected at home using standardized containers, delivered to the laboratory within a predefined time window and stored at −80 °C.

For metabolomic analysis, stool samples were removed from the −80 °C freezer and thawed on ice for 1 h. Thawed stool samples were collected using a pre-weighed FLOQ swab (Copan Diagnostics, Murrieta, CA 92562, USA, cat# 520CS01) into a homogenization vial (Benchmark Scientific, Sayreville, NJ 08872, USA, cat# D1031-T20), and the sample mass was measured. Samples were closed with aluminum foil and placed at −80 °C for 30 min prior to freeze-drying overnight. Dried stool samples were weighed, sealed with a screw cap, and kept at −80 °C until extraction. Wet weight, dry weight, and water content were calculated.

Urine samples were collected on the same day as stool whenever possible, using sterile containers, and processed according to a standardized protocol. After recording sample volume and creatinine concentration, aliquots were stored at −80 °C until metabolomic analysis.

### 4.4. Short-Chain Fatty Acid Analysis in Stool

SCFAs were quantified in stool extracts using a validated gas chromatographic method described by Adorno [97]. Briefly, stool samples were homogenized in tenfold amount (*w*/*w*) of milliQ (vigorous vortexing for 3 min, ultrasonication for 15 min and vortexing for 5 min). Following centrifugation (3270× *g*, 5 min, 10 °C), supernatant was used for diethyl ether extraction of SCFAs. Crotonic acid was used as internal standard. Gas chromatographic analysis was carried out on Agilent 6850N system with flame ionization detector (FID) (Agilent, Santa Clara, CA 95051, USA) equipped with a capillary column DB-WAX (122-7032E, J&W Scientific, Agilent, Santa Clara, CA 95051, USA).

The following SCFAs were measured: acetate (AC), propionate (PR), iso-butyrate (IB), butyrate (B), iso-valerate (IV), valerate (V), and caproate (CA). Concentrations were expressed in millimoles per liter of extract and then related to stool mass. In addition to absolute concentrations, total SCFAs (sum of all individual SCFAs) and derived indices (butyrate/acetate ratio, propionate/acetate ratio, and branched-chain SCFAs defined as IB + IV) were calculated. For normalization and sensitivity analyses, SCFA indices were standardized to stool dry weight.

### 4.5. Stool Tryptophan and Indole Metabolomics

Concentrations of tryptophan and its metabolites in stool were quantified using validated liquid chromatography–tandem mass spectrometry (LC–MS/MS) protocols adapted from previously published methods with minor modifications. Stool metabolites were analyzed as described. A defined volume of 80% isopropanol (IPA) was added to a known amount of stool, and the mixture was vortexed, sonicated, and centrifuged to obtain a clear extract. Metabolite concentrations were normalized using the NanoDrop-derived absorbance proxy and are reported as ng/a.u. for consistency across samples. This normalization was intended to reduce variability related to differences in extracted biological material and stool consistency across samples; however, it should be interpreted as a pragmatic proxy rather than a direct substitute for normalization to stool dry weight.

Using multiple reaction monitoring with stable isotope-labeled internal standards where available, we quantified the following metabolites: tryptophan (TRP), kynurenine (KYN), indole-3-acetic acid (IAA), indole-3-lactic acid (ILA), indole-3-propionic acid (IPA), indole-3-aldehyde (IAld), N-acetyl-tryptophan (NAc_TRP), serotonin (SER), melatonin (MEL), and tryptamine (TrpN). In addition, we derived functional ratios reflecting pathway balance, including KYN/TRP, IPA/TRP, IAA/TRP, SER/TRP, and an aggregate Indole/TRP ratio (sum of IAA, ILA, IPA, and IAld divided by TRP).

### 4.6. Urine Metabolomics

Urine metabolomics was performed using a standardized targeted platform [98]. In the present manuscript, urine data were used primarily to characterize multi-omics data availability and to support exploratory stool–urine association analyses, rather than to provide a full standalone urine metabolomics comparison.

### 4.7. Data Integration and Definition of Analysis Cohorts

All SCFA, stool metabolite, urine, and clinical data were integrated into a single master table, using the internal identifier AS xx to link records across laboratory and clinical files. For each child, the following information was compiled: group status (ASD vs. control), sex, age at sampling, CARS score (where available), SCFA profile, tryptophan-metabolite panel, and urine data availability. Only children with complete SCFA and stool tryptophan-panel data were included in the primary analysis cohort. Subset analyses were performed in the subgroup with available urine metabolomics data. Availability of 16S amplicon sequencing data was recorded as a binary variable to document multi-omics coverage for future microbiome–metabolite integration.

### 4.8. Statistical Analysis

All statistical analyses were performed using standard Statistical Software version 23.5.2 (MedCalc Software Ltd., Ostend, Belgium). Continuous variables are summarized as medians and interquartile ranges (IQRs) due to non-normal distributions. Group comparisons between ASD and controls for SCFA concentrations, tryptophan metabolites, and derived ratios were conducted using the Mann–Whitney U test. To control for multiple testing within each metabolite family (SCFAs and tryptophan-panel variables), *p*-values were adjusted using the Benjamini–Hochberg false discovery rate (FDR) procedure; adjusted q values < 0.05 were considered statistically significant.

Spearman correlation coefficients were calculated to explore the internal correlation structure among SCFAs and tryptophan metabolites and to quantify associations between stool metabolite indices and CARS scores within the ASD group. Correlation matrices and heatmaps were generated to visualize metabolic modules. In exploratory analyses, associations between selected stool metabolite indices and urine markers were evaluated using Spearman correlations in the subset with urine data.

## 5. Conclusions

In this well-characterized, school-age pediatric cohort, stool short-chain fatty acids and microbiota-derived tryptophan metabolites did not show robust cross-sectional differences between children with autism spectrum disorder and typically developing controls after correction for multiple testing. Moreover, these stool metabolites were not meaningfully associated with global ASD severity as measured by the Childhood Autism Rating Scale in the ASD subgroup. At the same time, the data revealed coherent and reproducible metabolic modules, with strongly intercorrelated SCFA indices and tryptophan-pathway variables, consistent with conserved microbiome-derived signaling axes relevant to host epigenetic and immune regulation.

Taken together, these findings suggest that gross alterations in stool SCFA and tryptophan profiles are unlikely to serve as standalone diagnostic markers for ASD in unselected school-age populations, but they support the existence of stable microbiota-derived metabolic programs that may still contribute to neurodevelopment through context-dependent and developmentally timed mechanisms.

## Figures and Tables

**Table 1:** Demographic and data-availability characteristics of the primary cohort (children with complete stool SCFA and tryptophan-metabolite data).

Variable	ASD (N = 160)	Controls (N = 69)	*p* Value
Age, years, median [IQR]	8.5 [6.1–12.4]	9.6 [6.4–13.2]	0.354
Male sex, n (%)	124 (77.5%)	39 (56.5%)	0.002
CARS score, median [IQR]	38 [30–44]	NA	–
CARS ≥ 30, n (%)	25 (73.5%)	NA	–
Clinical classification 1A, n (%)	12 (7.5%)	NA	–
Clinical classification 1B, n (%)	6 (3.8%)	NA	–
Clinical classification 2B, n (%)	35 (21.9%)	NA	–
16S amplicon sequencing available, n (%)	44 (27.5%)	50 (72.5%)	–
Stool SCFA data available, n (%)	160 (100.0%)	69 (100.0%)	–
Stool tryptophan panel available, n (%)	160 (100.0%)	69 (100.0%)	–
Urine metabolomics data available, n (%)	134 (83.8%)	60 (87.0%)	–

CARS, Childhood Autism Rating Scale; IQR, interquartile range; NA, not applicable.

**Table 2:** Stool short-chain fatty acids and derived indices in children with ASD and controls.

Variable	ASD Median	ASD Q25	ASD Q75	Control Median	Control Q25	Control Q75	*p* Value	q Value
Acetate (mM)	49.84	36.56	62.82	48.56	37.08	56.02	0.2853	0.3922
Propionate (mM)	12.67	8.98	17.96	14.06	10.73	17.23	0.1939	0.3751
Iso-butyrate (mM)	1.66	1.05	2.74	1.87	1.33	2.87	0.2046	0.3751
Butyrate (mM)	13.97	9.97	24.58	13.95	9.72	18.59	0.2689	0.3922
Iso-valerate (mM)	2.90	1.65	4.74	3.35	2.28	5.14	0.0987	0.3338
Valerate (mM)	1.59	1.04	2.61	2.03	1.11	2.57	0.5010	0.5511
Caproate (mM)	0.0	0.0	0.30	0.06	0.0	0.68	0.0426	0.2347
Total SCFAs (mM)	87.73	63.71	114.06	85.30	65.76	101.61	0.3595	0.4393
Butyrate/Acetate ratio	0.29	0.23	0.37	0.29	0.25	0.34	0.8389	0.8389
Propionate/Acetate ratio	0.27	0.21	0.32	0.30	0.24	0.35	0.0277	0.2347
Branched SCFAs (mM)	4.53	2.60	7.48	5.12	3.60	8.04	0.1214	0.3338

SCFAs, short-chain fatty acids.

**Table 3:** Stool tryptophan-pathway metabolites and ratios in children with ASD and controls. Normalized to absorption ratios (260/280 nm, 260/230 nm), a.u., arbitrary unit based on nucleic acid–proxy normalization.

Variable (ng/a.u.)	ASD Median	ASD Q25	ASD Q75	Control Median	Control Q25	Control Q75	*p* Value	q Value
Tryptophan	2.64	1.56	3.70	2.08	1.68	3.15	0.2213	0.7350
Kynurenine	2.17	1.46	3.84	2.06	1.35	4.34	0.9939	0.9939
Indole-3-acetic acid	57.24	24.31	104.08	46.80	21.82	94.90	0.6582	0.9089
Indole-3-lactic acid	18.78	8.05	37.79	15.14	7.93	30.91	0.3033	0.7350
Indole-3-propionic acid	343.46	157.51	657.45	272.81	172.69	412.32	0.1851	0.7350
Indole-3-aldehyde	151.85	86.05	297.63	144.23	69.55	239.27	0.2794	0.7350
N-acetyl-tryptophan	5.16	2.71	8.51	3.82	1.61	6.45	0.0223	0.3134
Serotonin	0.65	0.39	1.40	0.61	0.39	1.39	0.7616	0.9089
Tryptamine	1.03	0.50	2.43	0.84	0.46	2.79	0.7683	0.9089
KYN/TRP ratio	0.97	0.60	1.46	0.97	0.69	1.65	0.3675	0.7350
IPA/TRP ratio	127.68	60.84	243.62	119.79	83.18	201.06	0.8169	0.9089
IAA/TRP ratio	21.89	9.53	41.95	19.71	12.15	39.83	0.7833	0.9089
SER/TRP ratio	0.27	0.13	0.68	0.24	0.16	0.83	0.8440	0.9089
Indole/TRP ratio	261.18	183.23	467.61	243.16	184.18	389.44	0.3448	0.7350

a.u., arbitrary unit based on nucleic acid–proxy normalization; TRP, tryptophan; KYN, kynurenine; IPA, indole-3-propionic acid; IAA, indole-3-acetic acid; SER, serotonin.

**Table 4:** Spearman correlations between stool metabolite indices and CARS scores in children with ASD (n = 34).

Metabolite/Ratio	ρ with CARS	*p* Value
Butyrate	0.0652	0.7137
Propionate	0.0099	0.9555
Butyrate/Acetate	0.0833	0.6394
Propionate/Acetate	−0.0379	0.8314
KYN/TRP	0.0036	0.9835
IPA/TRP	0.1576	0.3733
Indole/TRP	0.1270	0.4740
SER/TRP	0.1446	0.4144

ρ, Spearman correlation coefficient; CARS, Childhood Autism Rating Scale.

## Data Availability

The data that support the findings of this study are available from the study’s principal investigator, J.O., upon reasonable request.

## Abbreviations

AC	acetate
AhR	aryl hydrocarbon receptor
ASD	autism spectrum disorder
a.u.	arbitrary unit (NanoDrop-based nucleic acid proxy normalization)
B	butyrate
CA	caproate
CARS	Childhood Autism Rating Scale
DSM-5	Diagnostic and Statistical Manual of Mental Disorders, 5th edition
ELISA	enzyme-linked immunosorbent assay
FDR	false discovery rate
FID	flame ionization detector
GC	gas chromatography
GI	gastrointestinal
HDAC	histone deacetylase
IAA	indole-3-acetic acid
IAld	indole-3-aldehyde
ILA	indole-3-lactic acid
IPA	indole-3-propionic acid
IQR	interquartile range
IV	iso-valerate
KYN	kynurenine
LC–MS/MS	liquid chromatography–tandem mass spectrometry
MEL	melatonin
MDC	microbiota-derived component
MDM	microbiota-derived metabolite
MIAOME	Microbiome Impact on the Host Epigenome
MSP	microbiota-secreted protein
NAc_TRP	N-acetyl-tryptophan
PR	propionate
SCFAs	short-chain fatty acids
SER	serotonin
TRP	tryptophan
TrpN	tryptamine
V	valerate
